# Factors Associated with the Participation of Older Adults in Cultural and Sports Activities

**DOI:** 10.3390/ijerph19106244

**Published:** 2022-05-20

**Authors:** Mihaela Ghența, Aniela Matei, Luise Mladen-Macovei, Elen-Silvana Bobârnat

**Affiliations:** 1 National Scientific Research Institute for Labour and Social Protection (INCSMPS), 010643 Bucharest, Romania; aalexandrescu@incsmps.ro (A.M.); luisemladen@hotmail.com (L.M.-M.); silvana.bobarnat@incsmps.ro (E.-S.B.); 2 Faculty of Sociology and Social Work, University of Bucharest, 030018 Bucharest, Romania

**Keywords:** older persons, cultural activities, sports activities

## Abstract

In the context of population aging, we have witnessed an increased interest in studying the participation of older persons in cultural and sport activities. The aim of this paper is to identify the participation rate in cultural and sports activities among Romanian older adults and the sociodemographics/behavioral variables that correlate with the participation rate in cultural and sports activities. In order to answer our research questions, we employed a questionnaire-based survey, and we used a nationally representative sample. Results point to low participation in both cultural and sports activities. Gender, residence, education, income, degree of mobility, internet connection, and availability of financial resources are significant variables that correlate with the engagement of older people in cultural and sports activities. Our study could serve as a base for concrete policy measures in the field of health and social inclusion of older persons.

## 1. Introduction

The aging of the population has generated an increased interest in studying the implication of cultural, sports, and physical activities of older persons in Europe. Participation in cultural and sports activities could contribute to the achievement of social inclusion at any age [1,2], with positive effects on health status, quality of life, and emotional wellbeing [3,4,5]. Although the effects of older people’s involvement in cultural and physical activity (including sport) are recognized as positive, their participation in such activities remains low, especially for those with low incomes or in less-developed communities [6,7].

While the number of the Romanian older persons is growing, the interest in cultural and sports activities remains low, not only among older Romanians but also for the overall population. The share of the aging population (aged 65+) reached almost 20% in 2021 and recorded a growing trend in the last decade [8]. Despite this aging decline and the proven benefits of participation in cultural and sports activities for individuals [6,7,9], little concern has been paid by policymakers to the wellbeing and quality of life of older persons.

The latest European data (2015) concerning older persons′ involvement in cultural activities point to a low rate of involvement: only 11.1% of persons 65–74 years old and 4.5% of those aged 75+ [10], and participation was even lower in rural areas [11]. In terms of most preferred cultural activity to participate in, older Romanians preferred to go to a museum or visit a historical site [11]. The main barriers to involvement mentioned by older Romanians are a lack of interest and limited choice [12]. With regard to sport activities, the latest European data (2017) [13] highlight that 63.3% of Romanians never participate in sports and also 79.2% of those aged 65+ never participate in sports. Other quantitative national studies also pointed to a low involvement in physical and sports activities and sedentary behaviors of Romanians as they age [14]. However, those that engage in sports or physical activities noted an improvement in health status (21.9%) and relaxation as the main reasons for engagement (18%) [13]. The main barriers to participation are a disability or an illness (27.1%), lack of motivation (14.3%), and lack of time (13.9%) [13].

Research at the national level with regard to the involvement of older persons in cultural and sports activities is scarce and represents attempts to understand the motivations, barriers, and behaviors of older persons [11,14,15].

The benefits of the involvement in cultural and sports activities are partially recognized by national health and public social policies but without concrete measures to raise awareness of the importance of such activities for the overall wellbeing of older persons.

The aim of this paper is to identify the variables—such as sociodemographics, mobility, ICT availability, and availability of financial resources that correlate with older adults′ participation in cultural and sports activities in Romania. Our research question is the following: “What are the variables that correlate with participation in cultural and sports activities?”

## 2. Literature Review

### 2.1. Cultural Activities

Older persons′ participation in cultural activities has received much attention in the scientific literature in terms of the types of activities preferred by this group of population, determinants of involvement, cultural needs, and effects of engagement and participation in such activities. The involvement of older persons in cultural activities could take the form of an active engagement (in this situation, older persons are directly involved as performers or creators) or a receptive involvement (in this situation, older persons are attenders of different forms of cultural activities) [16].

The benefits of cultural involvement of older persons are supported in terms of positive effects with regard to the physical and mental health of older adults and also in terms of contribution to the social inclusion of this population. Studies conducted in the field have highlighted that engagement in cultural activities reduces the risk of chronic pain [17] and plays an important role in the prevention, management, and treatment of different diseases [9]. Other studies concluded that involvement in cultural activities reduces the risk of depression in older age [16], the incidence of dementia [18], the incidence and progression of frailty [19], and improves the overall health of the participants [20,21].

Involvement in cultural activities contributes to the wellbeing of older persons [21,22,23,24] as it prevents or reduces the incidence of loneliness [20,25] as well as the odds of loneliness among older persons [26]. Cultural activities favor social connectedness and the quality and quantity of social relationships [25,26,27].

Decisions and forms of participation in cultural activities are related to individual factors, socioeconomic characteristics, and cultural context. Individual factors are the overall state of health (physical and mental) [28,29], needs [29], expectations [29], and lifestyle [29]. Other authors have argued that the social and cultural context and type of activities available influence the decision to engage in cultural activities [29]. Factors that contribute to the improvement of the social participation of older people are [29,30]: the area of residence; access to transport facilities, a transport network, or the availability of a specific means of transport that facilitates movement; social network; health status, which influences both the level of participation and the type of activities; caring responsibilities; and economic status. Other quantitative studies have highlighted the importance of parental education and the level of education of each individual [31] in their participation in cultural activities.

Differences between engagement and attendance of older persons to cultural activities were identified in the scientific literature with regard to barriers. Barriers to cultural engagement in old age are poor health status, a lack of time, and interest [32]. Studies in the field identified as the main barriers to participation in cultural activities, the socioeconomic and demographic characteristics of the individuals [33], health status, living arrangements, a low level of education [31], and economic resources available [31]. A lack of or high costs of transportation and inadequate transportation means were proved as barriers to participation in cultural activities [32]. Other quantitative studies [33] reported an association between geographical factors (the characteristics of the geographical area and neighborhood) and the cultural engagement of older adults.

### 2.2. Sport Activities

Physical activities imply the consumption of energy and include sports, exercises, or physical activities as part of one′s daily activities [34]. As a component of physical activity, sports are appreciated to have an important contribution to the social, cultural, and economic progress of individuals and nations [35,36]. The scientific literature studied older adults′ involvement in sports activities in terms of participation in both sport competition and community-based sports club events [35].

Positive effects of involvement in sports activities for older persons include the prevention and treatment of chronic disease [34], emotional and psychological imbalances and cognitive dysfunction [36,37], physical and mental health [38,39], and social health [39,40]. Social integration is also directly influenced by physical activities and sports participation, as they are one of the key factors for maintaining social connections and networks [39,40,41,42].

The benefits of older adults′ sports activities involvement [40] include intrapersonal benefits (related to social, physical, and mental health), interpersonal benefits (intergenerational connectivity within the family and role modeling), and organizational benefits (personal safety, flexibility of playing options).

A systematic review of quantitative and qualitative studies concerning the involvement of older adults in sports activities [5] identified the following factors as contributing to the involvement of older persons in sports activities: health status, negotiating the aging process through sport with two components—developing a positive aging discourse and overcoming negative stereotypes of aging, a previous history of participation in sports, interest in maintaining and developing social connections, and sociodemographic characteristics. Determinants of involvement in sports activities were grouped into health-related factors, social factors, relationship factors, competition/achievement factors, and successful aging factors [42].

Studies conducted in the field [43] identified the main barriers that limit the participation of older persons in physical/sports activities, which included poor health, a lack of a companion, a lack of adequate means of transport (perception of location accessibility proved to be important among elderly participants in the study), a lack of time available for such activities, and a lack of interest. In other qualitative studies [44], physical and mental health, socioemotional factors, and environmental context were identified as being both facilitators and barriers to older persons′ participation in sports activities, while family responsibilities (taking care of grandchildren) only represented a barrier. In other research, barriers to sports activities among older persons were grouped into intrapersonal factors (physical and mental health, skills), interpersonal factors (e.g., lack of time due to caring for grandchildren), and organizational factors (e.g., lack of information about sports programs, limited financial resources) [40].

## 3. Materials and Methods

### 3.1. Data Source and Sample Characteristics

To achieve the goals of this article, we used a survey database from the Innovative strategies for the promotion of social inclusion in later life, in the context of the societal challenges, financed through the Nucleu Programme and supported by the Romanian Ministry of Research, Innovation and Digitalization, project number PN 19130401.

The target population of the survey was represented by un-institutionalized Romanian people aged 65 and over, with the total volume of the sample being 802 adults aged 65+. The sampling error of the survey was ±3.5% for the national sample (Table 1).

A random stratified probabilistic sample was used with the following stratification criteria: (1) *development regions* (eight regions); (2) *locality type* (large urban, medium–small, and rural), and (3) *residential area* (urban, rural). Stratified random sampling is a probability sampling method using a two-step process to select the sample group. The population is first divided into homogeneous subpopulations, or strata, that are mutually exclusive and collectively exhaustive. This means that every element in the population must be assigned to only one stratum, and there should not be any overlap of elements across the strata [45,46,47].

In each county included in the sample, the selection of localities as primary sampling points was made randomly while respecting the stratification criteria mentioned above. In each locality, the polling stations were also randomly selected (as starting point for household selection). For the selection of households, the random route method was used based on a statistical step of 3 starting from the starting address (address of the polling station).

### 3.2. Recruitment and Data Collection

In the first phase, the questionnaire was pretested on 29 older adults from the target group; this stage was an important step in the survey [48,49] (Table 2). The second phase, data collection through nationally representative survey among older adults, took place between 11 and 27 November 2021. The rate of refusal to participate in the survey was around 25%, mainly due to fears about COVID-19, especially in urban areas (large urban).

The questionnaire was applied using the CAPI method (face-to-face interviews assisted by the operator and completed on the tablet). The application used was VoxCo (interview platform that also allows the application of questionnaires offline). The platform allows the recording of interview sequences as well as GPS coordinates to verify the quality of data collection. In this regard, respondents were asked, at the beginning of the interview, to agree on the quality control of data collection and processing of opinions, with all ethical issues regarding confidentiality, informed consent, and anonymity being addressed during the process of data collection.

### 3.3. Data Analysis Method

For the quantitative analysis of the data, we used IBM SPSS Statistics 23. To answer the research question, we used Chi-square test for independence, which is useful for exploring the statistical relationship between two categorical variables, regardless of the number of categories the variables have [50]. Before employing the analysis, we checked for the assumption referring to the lowest expected frequency [50], and we eliminated the variables in any cell that had an expected frequency of less than 5. First, we tested the statistical association between sociodemographics—such gender, residence (rural/urban), level of education, income level, and living alone status—and participation in cultural and sports activities, respectively. Second, we looked at variables related to mobility, such as the degree of mobility, the need for assistive devices, the accessibility of buildings, the accessibility of public places, and their association with the two dependent variables. Lastly, we considered the implications of internet connection and the availability of financial resources on participation in cultural and sports activities.

Our hypotheses were:

**Hypothesis** **1** **(H1):** *There is a statistically significant difference in the participation in cultural and sports activities due to sociodemographic variables.*

**Hypothesis** **2** **(H2):** *There is a statistically significant difference in the participation in cultural and sports activities due to mobility.*

**Hypothesis** **3** **(H3):** *There is a statistically significant difference in the participation in cultural and sports activities due to ICT availability.*

**Hypothesis** **4** **(H4):** *There is a statistically significant difference in the participation in cultural and sports activities due to the availability of financial resources.*

When interpreting the results, for the tables larger than 2 × 2 we used Chi-square test, and for 2 × 2 tables, we used Continuity Correction indicator, using the corresponding Asymp. Sig. (2-sided), according to [50] indications.

In order to measure the effect size of each variable on the attendance of cultural and sports activities, we used Phi and Cramer’s Test. We read Phi coefficient for 2 × 2 tables and Cramer’s coefficient for tables larger than 2 × 2, according to [50].

### 3.4. Description of the Variables

Participation in cultural and sports activities was measured with 2 items, 1 item for each type of activity. Sociodemographic section consisted of 5 items referring to gender, residence, level of education, income level, and living alone status. The mobility section consisted of 4 items referring to the degree of mobility, the need for assistive devices, the accessibility of buildings, and the accessibility of public spaces. The ICT availability section consisted of 1 item referring to internet connection availability. The availability of the financial resources had 2 items, the first asking about the availability of financial resources for basic needs and the second asking about the availability of financial resources for spare time expenses. We also included a section regarding the reasons for not participating in cultural and sports activities that consisted of 2 items, 1 for each type of activity. All questions were closed-ended questions. The full description of the variables used can be found in Appendix A, Table A1.

## 4. Results

### 4.1. The Participation in Cultural and Sports Activities

Of the 802 participants that gave valid responses, 90.3% (*n*: 723) had not attended cultural activities in the last 12 months, compared to 9.7% (*n*: 78) that had attended this type of activity in the last year. When looking at attendance in sports activities, the participation rate was even smaller, with 94.3% (*n*: 755) of the respondents declaring that they had not attended sports activities in the last year, while 5.7% (*n*: 46) had participated in this type of spare time activities in the same time frame (Appendix A, Table A2).

### 4.2. Sociodemographics

In terms of participation in cultural activities, statistically significant differences in terms of gender (Chi^2^ = 8.49, *p* < 0.01, phi = −0.107), residence (Chi^2^ = 4.20, *p* < 0.05, phi = −0.077), and level of education (Chi^2^ = 18.05, *p* = 0.00, Cramer’s = 0.150) were found (Appendix A, Table A3 and Table A5). An analysis of the adjusted values indicated that women (Adjusted Residual = 3) and people living in rural areas (Adjusted Residual = 2.2) are more likely to not engage in cultural activities compared to their counterparts. People with ISCED 0–2 education (Adjusted Residual = 2.8) are more likely to not participate in cultural activities compared to people with an ISCED 5–8 level of education (Adjusted Residual = 3.8), who are more likely to engage in this type of activity (Appendix A, Table A6).

In terms of participation in sports activities, statistically significant differences in terms of gender (Chi^2^ = 26.59, *p* = 0.00, phi = −0.188), residence (Chi^2^ = 7.85, *p* < 0.01, phi = −0.104), level of education (Chi^2^ = 23.68, *p* = 0.00, Cramer’s = 0.172), and level of income (Chi^2^ = 16.56, *p* = 0.00, phi = 0.154) were found (Appendix A, Table A4 and Table A5). An analysis of the adjusted values indicated that women (Adjusted Residual = 5.3), individuals living in rural areas (Adjusted Residual = 3), and people earning 364.28 euro or less (Adjusted Residual = 4.2) are more likely to not engage sports activities compared to their counterparts. People with ISCED 0–2 level of education (Adjusted Residual = 4.3) are more likely to not participate in sports activities, compared to people with ISCED 5–8 (Adjusted Residual = 3.3), who are more likely to participate in this type of activity (Appendix A, Table A6).

### 4.3. Mobility

Regarding the participation in cultural activities, statistically significant differences in terms of the degree of mobility (Chi^2^ = 5.57, *p* < 0.05, phi = 0.154) and the accessibility of public places (Chi^2^ = 4.37, *p* < 0.05, phi = 0.092) were found (Appendix A, Table A3 and Table A5). An analysis of the adjusted values indicated that people with a low degree of mobility (Adjusted Residual = 2.6) and people that live in environments with a low degree of accessibility to public places (Adjusted Residual = 2.2) are more likely to not engage in cultural activities compared to their counterparts (Appendix A, Table A6).

Regarding participation in sports activities, a statistically significant difference in terms of the degree of mobility (Chi^2^ = 4.39, *p* < 0.05, phi = 0.143) was found (Appendix A, Table A4 and Table A5). An analysis of the adjusted values indicated that individuals with low mobility (Adjusted Residual = 2.4) are more likely to not engage in sports activities compared to their counterparts (Appendix A, Table A6).

### 4.4. ICT Availability

ICT availability seems to be very important for cultural participation. Statistically significant difference in terms of internet connection (Chi^2^ = 13.28, *p* = 0.00, phi = −0.133) was found (Appendix A, Table A3 and Table A5). An analysis of the adjusted values indicated that people that do not have an internet connection (Adjusted Residual = 3.8) are more likely to not engage in cultural activities compared to their counterparts (Appendix A, Table A6).

ICT availability also looks to be important for sports participation. A statistically significant difference in terms of internet connection (Chi^2^ = 19.90, *p* = 0.00, phi = −0.163) was found (Appendix A, Table A4 and Table A5), with individuals without internet connection (Adjusted Residual = 4.6) being more likely to not engage sports activities compared to their counterparts (Appendix A, Table A6).

### 4.5. Availability of Financial Resources

In terms of participation in cultural activities, a statistically significant difference in terms of the availability of resources for spare time (Chi^2^ = 7.40, *p* < 0.05, Cramer’s = 0.110) was found (Appendix A, Table A3 and Table A5). People with low availability of financial resources for spare time (Adjusted Residual = 2.4) are more likely to not attend cultural activities compared to people with high availability of financial resources for spare time (Adjusted Residual = 2.5), who are more likely to attend cultural activities (Appendix A, Table A6).

When looking at the participation in sports activities, statistically significant differences in terms of both the availability of financial resources for basic needs (Chi^2^ = 7.96, *p* < 0.05, Cramer’s = 0.108) and the availability of financial resources for spare time activities (Chi^2^ = 32.97, *p* = 0.00, Cramer’s = 0.233) were found (Appendix A, Table A4 and Table A5). People with low availability of financial resources for basic needs (Adjusted Residual = 2.2) were more likely to not attend sports activities compared to people with high availability of financial resources for basic needs (Adjusted Residual = 2.6), who were more likely to attend these activities; people with low availability of financial resources for spare time (Adjusted Residual = 5.4) are more likely to not attend sports activities compared to people with medium and high availability of financial resources for spare time (Adjusted Residual = 2.3, Adjusted Residual = 4.8, respectively), who are more likely to attend these activities (Appendix A, Table A6).

### 4.6. The Reasons for Not Participating in Cultural and Sports Activities

When asked about their reasons for their non-participation in cultural activities, 29% of the respondents said they were not aware of any organized activities intended for older people, 33.6% declared there were no cultural activities in the area where they lived, 35.3% considered that their physical condition did not allow them to participate in cultural activities, 37.2% said that the pandemic context generated by COVID-19 was a barrier to attending cultural activities, 15.5% mentioned that their available financial resources did not allow them to attend cultural activities, and 11.8% mentioned other reasons, such as a lack of interest and lack of time (Appendix A, Table A7).

When asked about their reasons for their non-participation in sports activities, 26.4% of the respondents said they were not aware of any organized activities intended for older people, 28.3% declared that there were no sports activities in the area where they lived, 52.3% considered that their physical condition did not allow them to participate in sports activities, 31.4% said that the pandemic context generated by COVID-19 was a barrier to participation in sports, 9.5% mentioned that available their financial resources did not allow them to participate in sports activities, and 8.3% mentioned other reasons, such as a lack of interest and lack of time (Appendix A, Table A8).

## 5. Discussion

This paper explores how older persons′ participation in cultural and sports activities varies by sociodemographic characteristics, the degree of mobility, the need for assistive devices, the accessibility of buildings, the accessibility of public places, ICT availability, and the availability of the financial resources.

### 5.1. Participation in Cultural Activities

Our study shows that there is a small rate of participation of older Romanian persons in cultural activities, and they are thus being more prone to become socially isolated as they grow older, with negative effects on their overall quality of life. Compared to previous studies [11,14], due to the volume of the sample, the findings for this category of the population could be generalized to the overall older population. Comparable results concerning participation in cultural activities were observed in other studies from Central and East European countries, pointing to similar determinants of cultural involvement of older persons [29]. 

Regarding the *sociodemographic characteristics*, our findings showed that participation in cultural activities is dependent on gender, residence area, and educational level, while income level and living alone status were found to be independent of participation of older persons in cultural activities. In our study, *gender* is a variable that correlates with non-participation in cultural activities, and women are more likely to not involve themselves in cultural activities. Studies [51] conducted on persons aged 60+ measuring the impact of cultural and social involvement on wellbeing pointed out that women who did not participate in social and cultural activities had a lower perception of wellbeing and resilience compared to men. Other studies [52] reported different results, with women (aged 65+) being more likely to desire to be involved in different social activities (including cultural activities) compared to men. Older persons from *rural areas* were also more likely to not participate in cultural activities. In rural settings, there are fewer cultural services compared to urban areas [29]. The availability and access to cultural activities create the conditions for cultural engagement and favor a healthy lifestyle among older persons [51]. Other studies found that older adults living in rural settings were less socially involved compared to those from urban areas [52], as despite the aging of the population, less is known about the needs of older persons in rural areas [53,54]. Existing studies pointed out that transportation and limited information about available activities lowered the social participation of people living in rural areas [54]. Different results were found in [33], as older persons who lived in rural areas registered higher odds of being culturally engaged compared to those from urban areas. In the same study, not only the type of area of residence but also the neighborhood characteristics influenced people′s involvement in cultural activities. Respondents with a low educational level (ISCED 0–2) were more likely to not become involved in cultural activities. Similar findings were found in existing studies [29,31]. The level of income is a determinant of participation in cultural activities [31], as well as the existence of a partner, children, or friends [27]. In this regard, our results seem to point to the need for further research to obtain a deeper understanding of the reasons for non-participation in cultural activities.

With respect *to mobility*, the degree of mobility and the accessibility of public spaces were related to participation in cultural activities, while the accessibility of buildings and the need for assistive devices were found to be unrelated to cultural participation in old age. The lack of accessibility of public spaces restricts the participation of older persons not only in cultural events but in the entire community life; thus, urban and rural settings need adaptations in order to be in line with the needs of an older population [32,55]. People with a low degree of mobility are less interested in participating in cultural activities. Previous studies concluded that mobility is an important determinant of older persons′ engagement in outdoor activities [56]. The use of assistive devices may increase participation in cultural activities, as other studies have demonstrated [57]. Considering other national research [14], the low participation rate may be related to sedentary behavior among the older population.

Regarding the *access to an internet connection*, our findings show that this variable correlates with participation in cultural activities, as respondents who have no internet connection are more likely to not be engaged in cultural activities. Qualitative studies [58] pointed out that internet connections facilitate access to information related to cultural events and create opportunities for older adults to participate.

Respondents with low availability of *financial resources* for spare time were more likely to not participate in cultural activities, according to our results. In this respect, our findings are in line with the studies that emphasize a relationship between the availability of income for different types of activities and participation in cultural activities [30,59].

### 5.2. Participation in Sports Activities

The low participation of the older adults in sports activities resulting from the analysis of statistical data [13] is also confirmed by the results obtained within our survey. Scientific studies highlight a gender gap in participation in sports activities, showing that women are less regularly involved in such activities compared to men [60], which is in line with our findings.

In relation to *sociodemographic characteristics*, our study found that participation in sports activities is dependent on gender, residence area, level of education, and income level. At the same time, there seems to be no association between the participation of older people in sports activities and living alone status. However, there are studies that highlight the fact that people living alone are more likely to have lower physical activity levels than their married peers [61].

*Gender* differences emerged as a determinant in participation in both quantitative [62] and qualitative studies [63]. Recent papers, however, document a more ambiguous picture of gender differences regarding sports participation in general and specifically with respect to older persons. While some studies identify no significant gender difference in participation rates in sports for older adults, other studies show a significantly higher participation rate of either men in some age groups 50+ or women 50+ [64].

A number of studies have shown a positive association between the participation of older adults in sports and their *educational level* [64,65,66]. We have also found that people with an ISCED 0–2 level of education are more likely to not participate in sports activities compared to people with ISCED 5–8, who are more likely to participate in this type of activity.

Our research has also shown that people with *low incomes* are less likely to participate in sports. Older adults with lower incomes do not have enough money to facilitate their time management and inclusion in physical activities. This finding aligns with the previous literature, which showed that those who had a higher socioeconomic status were more likely to engage in physical activities [67,68]. There are qualitative studies that found that financial constraints on the affordability of exercise equipment, the commitment of individuals to participate in sports, and the perceived stigma for being below the poverty line were barriers that caused low participation in sports and physical activities [69]. Indeed, some sports are expensive and less accessible for people on low incomes, but walking, for example, does not cost anything. The level of people’s involvement in sport can also probably be explained by other factors that are not directly financial but are associated with higher income, e.g., higher educational attainment, social background and position, greater awareness of the benefits of physical activity, more free time, etc. 

Sports are an urban phenomenon. Participation in sports is higher in urban areas compared to rural areas [70]. Our paper also highlighted that older people in *rural areas* are less likely to participate in sports.

European and national sports participation surveys have shown that the practice of outdoor activities in non-competitive, informal groups with aims to improve or maintain health is growing in all EU countries. Public spaces such as streets, parks, and mountains are favored locations to play sports in most countries [71]. We found statistically significant differences related to participation in sports activities by the degree of *mobility*, but no association between participation in this kind of activities and the need for assistive devices, buildings’ accessibility, or public places’ accessibility. Our study showed that people who have a low degree of mobility are less likely to engage in sports activities, and this confirms what other studies concluded regarding this aspect [56]. There are, however, studies that indicate that neighborhood characteristics such as access to green spaces/shops, pedestrian-friendly features, and esthetically pleasing scenery positively affected older adults’ physical activity participation [72,73].

*Access to the internet* correlates with participation in sports activities. Respondents who do not have an internet connection are more likely to not engage in sports activities compared to those who have one. There are several studies, both quantitative [70] and qualitative [74], that reveal the positive association between access to the internet and participation in recreational physical activity or sport. Therefore, our results can be considered as validated.

With respect to *financial resources*, we find an association between participation in sports activities and the availability of resources for basic needs and resources for spare time. This finding is in line with other studies that highlight the importance of economic resources for an active life and involvement in sports and recreational physical activities [59].

### 5.3. Limitations of the Research

This paper has a number of weaknesses. Although it identifies the variables that correlate with non-participation in sports and cultural activities and uses an ordinal scale to measure the frequency of participation (weekly, monthly, several times in the last 12 months, and one time in the last 12 months), the small number of those that participated in cultural and sports activities did not allow us to conduct further analyses. The health status of the respondents was not assessed during the survey, and the correlation of this variable with the participation in cultural and sports activities could not be assessed. Although we identified the variables that correlate with participation in cultural and sports activities, we still have no understanding of the reasons for which the participation in cultural and sports activities of older persons is low, and this should be investigated in future research.

Despite the limitations, this study has a number of strengths. We should underline that we used a nationally representative sample (covering adults 65+ from both urban and rural areas) and a set of variables that allow comprehending older individuals’ participation in sports and cultural activities. The results and the conclusions could be extended to the whole population aged 65+ in Romania. The volume of the sample size allowed us to group participants by their educational attainment (three sub-groups).

Considering the scarcity of national studies dedicated to the understanding of older persons’ decisions in this regard and the fact that studies available approach participation with regard to only one type of leisure activity (e.g., cultural, physical, sport) or only for older persons that reside in urban areas, we consider that this paper deepens the knowledge in this field and provides information to substantiate active aging and health policies. Future research should further the understanding of the factors that determine the participation in cultural and sport/physical activities of older persons and ways to increase the participation in such activities.

## 6. Conclusions

Our study showed that in Romania, there is a small participation rate of older persons in cultural and sports activities.

In terms of participation in cultural activities, data analysis indicated that women, people living in rural areas, people with a low degree of mobility, people that live in environments with low accessibility to public places, and people who do not have an internet connection are more likely to not engage in cultural activities compared to their counterparts. It was also found that people with an ISCED 0–2 education are more likely to not participate in cultural activities compared to people with an ISCED 5–8 level of education. Additionally, people with low availability of financial resources for spare time are more likely to not attend cultural activities compared to people with high availability of financial resources for spare time, who are more likely to attend cultural activities.

Regarding the participation in sports activities, an analysis of the survey data indicated that women, individuals living in rural areas, people with low earnings, individuals with low mobility, and individuals without an internet connection are more likely to not engage in sports activities compared to their counterparts. People with an ISCED 0–2 level of education are more likely to not participate in sports activities compared to people with an ISCED 5–8, who are more likely to participate in this type of activity. At the same time, people with low availability of financial resources for basic needs and people with low availability of financial resources for spare time are more likely to not attend sports activities.

The main three reasons for non-participation in cultural activities were, in order, the pandemic context generated by COVID-19, the physical condition of the respondent, and the lack of availability of cultural activities in the area where the older person lives. The main reason for non-participation in sports activities was the physical condition of the respondent, followed by the pandemic context and then the lack of availability of sports activities in the area.

All the results obtained in our research support the current knowledge on factors that determine the participation of older persons in cultural and sports activities and serve as evidence to substantiate health and social policies for older adults.

## Figures and Tables

**Table 1:** Main sociodemographic characteristics of the respondents included in the survey sample.

Name of the Characteristic	Total (*n* = 802)	Total %
**Sex**		
*Male*	344	42.9
*Female*	458	57.1
**Age Category**		
*65–74 years old*	561	70.0
*75–84 years old*	202	25.2
*Over 85 years old*	39	4.9
**Residential Area**		
*Urban*	442	55.1
*Rural*	360	44.9
**Last Educational Level**		
*ISCED 0–2*	246	30.7
*ISCED 3–4*	444	55.4
*ISCED 5–8*	112	14.0
**Income Level**		
*364.28 euro (1800 RON) or less*	483	63.9
*Over 364.28 euro (1800 RON)*	273	36.1
**Living Alone Status**		
*Living alone*	283	35.3
*Not living alone*	519	64.7

Source: Developed by authors based on the methodological report of the survey.

**Table 2:** Phases of the research field.

Phase	Total (*n* = 802)	Means
**A. Pretesting of the questionnaire** *Data analysis* *Recalibration of the questionnaire*	*n* = 29	IBM SPSS 21
**B. Data collection through nationally representative survey among older adults aged 65+**	*n* = 802	IBM SPSS 21

Source: Developed by authors based on the methodological report of the survey.

## Data Availability

Not applicable.

## Appendix A

**Table A1:** The description of the variables.

Variables	Description
Participation in cultural and sports activities	
Participation in cultural activities	“In the last 12 months, have you participated in cultural activities (e.g., visiting a museum, watching a concert, a movie, watching a show)?” The variable is binary, and the “Yes” responses were coded as 1, and the “No” responses were coded as 0.
Participation in sports activities	“In the last 12 months, have you participated in sports activities (for example, light sports activities, sports competitions, etc.)?” The variable is binary and the “Yes” responses were coded as 1, and the “No” responses were coded as 0.
Sociodemographics	
Gender	Gender is a binary variable that takes the value 1 in the case of male respondents and 0 for female respondents.
Residence	The residence is also an alternative variable, where 1 represents an urban residence and 0 is a rural residence.
Level of education	The level of education is categorical variable with three possible values: 1 for ISCED 0–2, 2 for ISCED 3–4, and 3 for ISCED 5–8.
Income level	The income level is a binary variable that takes the value 0 for people earning 364.28 euros (1 euro = 4.9412 RON, 1800 RON in Romanian currency) and value of 1 for people earning more than 364.28 RON (this being the average pension for the age limit).
Living alone status	The living alone status is also a binary variable that takes value of 0 for respondents living alone and value of 1 for respondents living with somebody else.
Mobility	
The degree of mobility	The degree of mobility is a binary variable that takes value of 0 for respondents that have low mobility, inside their community, outside their community, or both, and value of 1 for respondents with high mobility.
The need for assistive devices	The need for assistive devices is a binary variable that takes value of 0 for the respondents who mentioned that they need assistive devices and 1 for the respondents who specifically mentioned that they do not need this type of device.
The accessibility of buildings	The accessibility of buildings is a binary variable that takes value of 0 for low accessibility and 1 for high accessibility.
The accessibility of public places	The accessibility of public places is a binary variable that takes value of 0 for low accessibility and 1 for high accessibility.
ICT availability	
ICT availability	Internet connection is also a binary variable that takes value of 0 for the cases in which the respondents have no internet connection and 1 for the opposite situation, when the respondents have an internet connection.
The availability of financial resources	
The availability of financial resources for basic needs	The availability of financial resources for basic needs (food, medical treatment, clothes and shoes, utilities, house maintenance) is a categorical variable that takes one of the 3 values: 1 for low, 2 for medium, and 3 for high availability of the financial resources for basic needs.
The availability of financial resources for spare time expenses	The availability of financial resources for spare time expenses (travel, transport, leisure, and sports activities, cultural activities like shows, exhibitions, reading books and magazines) is also a categorical variable that takes 1 of three values: 1 for low, 2 for medium, and 3 for high availability of the financial resources for spare time expenses.
The reasons for not participating in cultural and sports activities	
The reasons for not attending cultural activities	The questions were multiple-responses questions with the following choices: “I was not aware of any organized activities intended for the older people”, “There are no cultural/sports activities in the area where I live”, “My physical condition does not allow me to participate in cultural/sports activities”, “The pandemic context generated by COVID-19 was a barrier in attending cultural/sports activities”, “The available financial resources do not allow me to attend cultural/sports activities”, “Another reason (which other reason?)”.
The reasons for not attending sports activities	

**Table A2:** Distribution of the participation in cultural and sports activities in the last 12 months.

	Cultural Activities		Sports Activities	
	***n***	**%**	***n***	**%**
No	723	90.3%	755	94.3%
Yes	78	9.7%	46	5.7%

Source: Developed by the authors based on the survey data.

**Table A3:** Cultural activities participation in the last 12 months by sociodemographics, mobility, ICT accessibility, and availability of financial resources.

			Attendance of Cultural Activities	
			No	Yes
*Sociodemographics*				
Gender	Female	*n*	426	32
		%	93.0%	7%
	Male	*n*	297	46
		%	86.6%	13.4%
	Chi^2^ = 8.49, *p* < 0.01			
Residence	Urban	*n*	389	52
		%	88.2%	11.8%
	Rural	*n*	334	26
		%	92.8%	7.2%
	Chi^2^ = 4.20, *p* < 0.05			
Level of education	ISCED 0–2	*n*	233	13
		%	94.7%	5.3%
	ISCED 3–4	*n*	400	43
		%	90.3%	9.7%
	ISCED 5–8	*n*	90	22
		%	80.4%	19.6%
	Chi^2^ = 18.05, *p* = 0.00			
Income level	364.28 euros (1800 RON) or less	*n*	442	40
		%	91.7%	8.3%
	Over 364.28 euros (1800 RON)	*n*	239	34
		%	87.5%	12.5%
	Chi^2^ = 2.95, *p* = 0.09			
Living alone status	Yes	*n*	257	26
		%	90.8%	9.2%
	No	*n*	466	52
		%	90.0%	10.0%
	Chi^2^ = 0.07, *p* = 0.79			
*Mobility*				
Degree of mobility	Low	*n*	88	4
		%	95.7%	4.3%
	High	*n*	157	27
		%	85.3%	14.7%
	Chi^2^ = 5.57, *p* < 0.05			
Need for assistive devices	Yes	*n*	95	6
		%	94.1%	5.9%
	No	*n*	628	72
		%	89.7%	10.3%
	Chi^2^ = 1.43, *p* = 0.231			
Buildings’ accessibility	Low	*n*	286	22
		%	92.9%	7.1%
	High	*n*	382	48
		%	88.8%	11.2%
	Chi^2^ = 2.23, *p* = 0.087			
Public places’ accessibility	Low	*n*	219	17
		%	92.8%	7.2%
	High	*n*	301	45
		%	87.0%	13.0%
	Chi^2^ = 4.37, *p* < 0.05			
*ICT accessibility*				
Internet connection	Yes	*n*	243	43
		%	93.2%	6.8%
	No	*n*	480	35
		%	85.0%	15.3%
	Chi^2^ = 13.28, *p* = 0.00			
*Financial resources availability*				
Resources for basic needs expenses	Low	*n*	226	19
		%	92.2%	7.8%
	Medium	*n*	212	33
		%	86.5%	13.5%
	High	*n*	177	20
		%	89.8%	10.2%
	Chi^2^ = 4.30, *p* = 0.117			
Resources for spare time expenses	Low	*n*	389	40
		%	90.7%	9.3%
	Medium	*n*	83	13
		%	80.7%	19.3%
	High	*n*	67	16
		%	88.7%	11.3%
	Chi^2^ = 7.40, *p* < 0.05			

Source: Developed by the authors based on the survey data.

**Table A4:** Sports activities participation in the last 12 months by sociodemographics, mobility, ICT accessibility, and availability of financial resources.

			Attendance of Sports Activities	
			No	Yes
*Sociodemographics*				
Gender	Female	*n*	449	9
		%	98.0%	2%
	Male	*n*	306	37
		%	89.2%	10.8%
	Chi^2^ = 26.59, *p* = 0.00			
Residence	Urban	*n*	406	35
		%	92.1%	7.9%
	Rural	*n*	349	11
		%	96.9%	3.1%
	Chi^2^ = 7.85, *p* < 0.01			
Level of education	ISCED 0–2	*n*	245	1
		%	99.6%	0.4%
	ISCED 3–4	*n*	412	31
		%	93.0%	7.1%
	ISCED 5–8	*n*	98	14
		%	87.5%	12.5%
	Chi^2^ = 23.68, *p* = 0.00			
Income level	364.28 euros (1800 RON) or less	*n*	468	14
		%	97.1%	2.9%
	Over 364.28 euros (1800 RON)	*n*	245	28
		%	89.7%	10.3%
	Chi^2^ = 16.56, *p* = 0.00			
Living alone status	Yes	*n*	270	13
		%	95.4%	4.6%
	No	*n*	485	33
		%	93.6%	6.4%
	Chi^2^ = 0.77, *p* = 0.382			
*Mobility*				
Degree of mobility	Low	*n*	91	1
		%	98.9%	1.1%
	High	*n*	169	15
		%	91.8%	8.2%
	Chi^2^ = 4.39, *p* < 0.05			
Need for assistive devices	Yes	*n*	99	2
		%	98.0%	2.0%
	No	*n*	656	44
		%	93.7%	6.3%
	Chi^2^ = 2.28, *p* = 0.131			
Buildings’ accessibility	Low	*n*	295	13
		%	95.8%	4.2%
	High	*n*	397	33
		%	92.3%	7.7%
	Chi^2^ = 3.10, *p* = 0.079			
Public places’ accessibility	Low	*n*	227	9
		%	96.2%	3.8%
	High	*n*	319	27
		%	92.2%	7.8%
	Chi^2^ = 3.19, *p* = 0.074			
*ICT accessibility*				
Internet connection	Yes	*n*	255	31
		%	97.1%	2.9%
	No	*n*	500	15
		%	89.2%	10.8%
	Chi^2^ = 19.90, *p* = 0.00			
*Financial resources availability*				
Resources for basic needs expenses	Low	*n*	237	8
		%	96.7%	3.3%
	Medium	*n*	231	14
		%	94.3%	5.7%
	High	*n*	178	19
		%	90.4%	9.6%
	Chi^2^ = 7.96, *p* < 0.05			
Resources for spare time expenses	Low	*n*	417	12
		%	97.2%	2.8%
	Medium	*n*	85	11
		%	88.5%	11.5%
	High	*n*	68	15
		%	81.9%	18.1%
	Chi^2^ = 32.97, *p* = 0.00			

Source: Developed by the authors based on the survey data.

**Table A5:** Intensity of correlations between social participation in the last 12 months and sociodemographics, mobility, ICT accessibility, and availability of financial resources.

	Attendance of Cultural Activities	Attendance of Sports Activities
*Sociodemographics*		
Gender	−0.107 **	−0.188 *
Residence	−0.077 **	−0.104 *
Level of education	0.150 *	0.172 *
Income level	no sig. diff.	0.154 *
Living alone status	no sig. diff.	no sig. diff.
*Mobility*		
Degree of mobility	0.154 **	0.143 **
Need for assistive devices	no sig. diff.	no sig. diff.
Buildings’ accessibility	no sig. diff.	no sig. diff.
Public places’ accessibility	0.092 **	no sig. diff.
*ICT accessibility*		
Internet connection	−0.133 *	−0.163 *
*Financial resources availability*		
Resources for basic needs expenses	no sig. diff.	0.108 **
Resources for spare time expenses	0.110 **	0.233 *

Phi and Cramer’s V Test, * *p* < 0.01, ** *p* < 0.05. Source: Developed by the authors based on the survey data.

**Table A6:** Probability of social participation in the last 12 months by sociodemographics, mobility, ICT accessibility, and availability of financial resources.

		Cultural Activities—Adjusted Residual		Sports Activities—Adjusted Residual	
		No	Yes	No	Yes
*Sociodemographics*					
Gender	Female	3.0	−3.0	5.3	−5.3
	Male	−3.0	3.0	−5.3	5.3
Residence	Urban	−2.2	2.2	−3.0	3.0
	Rural	2.2	−2.2	3.0	−3.0
Level of education	ISCED 0–2	2.8	−2.8	4.3	−4.3
	ISCED 3–4	0	0	−1.7	1.7
	ISCED 5–8	−3.8	3.8	−3.3	3.3
Income level	364.28 euros (1800 RON) or less	1.8	−1.8	4.2	−4.2
	Over 364.28 euros (1800 RON)	−1.8	1.8	−4.2	4.2
Living alone status	Yes	0.4	−0.4	1.0	−1.0
	No	−0.4	0.4	−1.0	1.0
*Mobility*					
Degree of mobility	Low	2.6	−2.6	2.4	−2.4
	High	−2.6	2.6	−2.4	2.4
Need for assistive devices	Yes	1.4	−1.4	1.7	−1.7
	No	−1.4	1.4	−1.7	1.7
Buildings′ accessibility	Low	1.8	−1.8	1.9	−1.9
	High	−1.8	1.8	−1.9	1.9
Public places′ accessibility	Low	2.2	−2.2	2.0	−2.0
	High	−2.2	2.2	−2.0	2.0
*ICT accessibility*					
Internet connection	Yes	−3.8	3.8	−4.6	4.6
	No	3.8	−3.8	4.6	−4.6
*Financial resources availability*					
Resources for basic needs expenses	Low	1.7	−1.7	2.2	−2.2
	Medium	−1.9	1.9	0.2	−0.2
	High	0.2	−0.2	−2.6	2.6
Resources for spare time expenses	Low	2.4	−2.4	5.4	−5.4
	Medium	−0.7	0.7	−2.3	2.3
	High	−2.5	2.5	−4.8	4.8

Source: Developed by the authors based on the survey data.

**Table A7:** Reasons for non-participation in cultural activities.

Reasons for Non-Participation in Cultural Activities	Percentage of Cases
I was not aware of any organized activities intended for the older people	29.0%
There are no cultural activities in the area where I live	33.6%
My physical condition does not allow me to participate in cultural activities	35.3%
The pandemic context generated by COVID-19 was a barrier to attending cultural activities	37.2%
The available financial resources do not allow me to attend cultural activities	15.5%
Another reason (which other reason?)	11.8%

Source: Developed by the authors based on the survey data.

**Table A8:** Reasons for non-participation in sports activities.

Reasons for Non-Participation in Sports Activities	Percentage of Cases
I was not aware of any organized activities intended for the older people	26.4%
There are no sports activities in the area where I live	28.3%
My physical condition does not allow me to participate in sports activities	52.3%
The pandemic context generated by COVID-19 was a barrier to attending sports activities	31.4%
The available financial resources do not allow me to attend sports activities	9.5%
Another reason (which other reason?)	8.3%

Source: Developed by the authors based on the survey data.
